# The Co-Repressor SMRT Delays DNA Damage-Induced Caspase Activation by Repressing Pro-Apoptotic Genes and Modulating the Dynamics of Checkpoint Kinase 2 Activation

**DOI:** 10.1371/journal.pone.0059986

**Published:** 2013-05-17

**Authors:** Claudio Scafoglio, Marcus Smolka, Huilin Zhou, Valentina Perissi, Michael G. Rosenfeld

**Affiliations:** 1 Howard Hughes Medical Institute and School of Medicine, University of California San Diego, La Jolla, California, United States of America; 2 Department of Molecular and Medical Pharmacology, David Geffen School of Medicine, University of California Los Angeles, Los Angeles, California, United States of America; 3 Ludwig Institute for Cancer Research and Department of Cellular and Molecular Medicine, University of California San Diego, La Jolla, California, United States of America; 4 Weill Institute for Cell and Molecular Biology, Cornell University, Ithaca, New York, United States of America; 5 School of Medicine, Boston University, Boston, Massachusetts, United States of America; Roswell Park Cancer Institute, United States of America

## Abstract

Checkpoint kinase 2 (Chk2) is a major regulator of DNA damage response and can induce alternative cellular responses: cell cycle arrest and DNA repair or programmed cell death. Here, we report the identification of a new role of Chk2 in transcriptional regulation that also contributes to modulating the balance between survival and apoptosis following DNA damage. We found that Chk2 interacts with members of the NCoR/SMRT transcriptional co-regulator complexes and serves as a functional component of the repressor complex, being required for recruitment of SMRT on the promoter of pro-apoptotic genes upon DNA damage. Thus, the co-repressor SMRT exerts a critical protective action against genotoxic stress-induced caspase activation, repressing a functionally important cohort of pro-apoptotic genes. Amongst them, SMRT is responsible for basal repression of *Wip1*, a phosphatase that de-phosphorylates and inactivates Chk2, thus affecting a feedback loop responsible for licensing the correct timing of Chk2 activation and the proper execution of the DNA repair process.

## Introduction

The induction of DNA damage by environmental carcinogens induces a very complex response aimed at repairing the alterations in the DNA structure and sequence [1], [2]. The detection of base modifications or strand breaks by sensor proteins induces the activation of a checkpoint response that allows the cell to block proliferation and to repair DNA [3]–[5]. If the damage is too extensive to be reversed, checkpoint proteins induce a switch from repair to programmed cell death, thereby preventing the transmission of mutations to the next cellular generations [6]. Caspase activation is a central event in the induction of apoptosis after DNA damage as well as other stimuli [6]. The mechanisms by which a cell discriminates between reparable damage and lethal genotoxic stress leading to caspase activation and apoptosis are not well understood.

Checkpoint kinase 2 (Chk2) is a central DNA damage checkpoint regulator [7], [8], responding mostly to stimuli that cause double strand breaks, such as ionizing radiation or topoisomerase II inhibitors such as doxorubicin. The kinase ATM is involved in sensing the damage and initiating the checkpoint response [9]. Among other substrates, ATM phosphorylates Chk2 on threonine 68 [10], [11], inducing dimerization of Chk2 and auto-phosphorylation of multiple sites, leading to full activation [12]–[15]. Chk2 activation results in cell cycle arrest [16] and activation of the DNA repair process [17], [18]. Finally, if the damage is too extensive to be repaired, Chk2 is able to induce apoptosis, through phosphorylation of the pro-apoptotic transcription factors p53 [19], [20] and E2F1 [21].

It has been shown that the ATM-Chk2-p53 system is activated with an oscillatory pattern, as a consequence of the equilibrium between activating stimulus (DNA damage) and a double feedback system activated by p53 and including Mdm2, which induces p53 degradation [22], and Wip1, a protein phosphatase that dephosphorylates both ATM and Chk2 [23]. The oscillations are considered a mechanism to check on the progress of the DNA damage and shut down the checkpoint response if the repair has been successful [24]. It has also been proposed that the oscillatory activation of p53 provides a means to fine-tune the cellular response and produce different outcomes according to the severity of the DNA damage [25].

Besides p53, other transcription factors play different roles in modulating the apoptotic response to DNA damage. Among these, NFκB and AP1 have a fundamental function. NFκB shows preeminent pro-survival actions in non-lymphoid tissues [26]–[29]; however, it has been shown that DNA damaging stimuli such as UV-C and doxorubicin/daunorubicin induce a switch of NFκB action from activator to repressor on the anti-apoptotic genes X-IAP, Bcl-X(L) and survivin, thus contributing to the induction of programmed cell death [30].

AP1 has been associated with regulation of apoptosis in response to a wide variety of stimuli, such as growth factor deprivation and environmental stresses, including DNA damage [31], [32]. The pro-apoptotic action is mediated by the transcriptional activation of FasL, that triggers the extrinsic apoptotic pathway [33], as well as Bim [34]. AP1-mediated repression also plays an important role in regulating the apoptotic switch: proteins of the AP-1 family are able to repress pro-apoptotic genes such as p53 [35], [36] and Fas [37]; therefore, the final outcome depends on the balance between gene activation and repression, and is highly influenced by several considerations including cell type, conditions of growth, and the presence of growth factors [38]. It is generally accepted that DNA damage activates initially both pro-apoptotic and anti-apoptotic pathways, each one subjected to subtle and complex regulation by different stimuli and transcription factors, and that the overall balance between genes promoting survival and genes inducing death signals dictates the cellular outcome [31].

Transcriptional co-repressors, recruited by transcription factors bound on DNA regulatory elements, act as a platform for further recruitment of repressive proteins including histone deacetylases or methyltransferases, which locally modify the structure of chromatin in a way that restricts access to activator complexes and RNA polymerases [39]. The core NCoR/SMRT co-repressor complexes contain the histone deacetylase HDAC3 [40], the exchange factors TBL1 and TBLR1 [41]–[43], and the G-protein pathway suppressor, GPS2 [44]. The NCoR/SMRT complexes are required for basal repression of genes by unliganded nuclear receptors, such as thyroid hormone receptor (TR) and retinoic acid receptor (RAR) [43], or for active repression mediated by ligand-bound receptors, such as tamoxifen-bound estrogen receptor [45]. NCoR/SMRT have also been associated with repression by a number of other transcription factors, including the regulators of apoptosis NFκB [46] and AP1 [47].

Although sharing significant sequence and structure homology and the interaction with members of the respective co-repressor complexes [43], [48], [49], NCoR and SMRT have been shown to play distinct and specific roles in differentiation and development [50]–[52], and to be subjected to different regulatory pathways [51], [53], [54].

Here we report the finding that, upon DNA damage, Chk2 is required to specifically recruit the co-repressor SMRT to repress pro-apoptotic genes, including AP1 and NFκB targets, thus keeping the apoptotic response on hold, while the cells repair the damage. In particular, SMRT-dependent repression of the protein phosphatase *Wip1*, a major regulator of checkpoint response that de-phosphorylates Chk2, ATM, p53 and p38 [55], is fundamental for caspase activation after DNA damage by modulating the oscillatory frequency of Chk2 activation.

## Results

### Chk2 interacts with SMRT and regulates different transcription factors, acting as a repressor

To gain insight into the role of the checkpoint kinase Chk2 in the regulation of the apoptotic switch after DNA damage [19], [56], [57], we first sought to identify the protein complex associated with Chk2. To this end the protein interaction domain (FHA domain) of human Chk2 was expressed in bacteria and the purified protein incubated with HeLa cell extract. This approach identified, amongst others, TBLR1, a component of the NCoR/SMRT co-repressor complexes, as a Chk2 interacting protein (Figure S1A–B). To confirm the interaction, flag-tagged full-length human Chk2 was expressed both in HeLa (data not shown) and U2OS cells and anti-Flag antibody was used to immunoprecipitate associated proteins, followed by Western blot analysis with antibodies specific for members of the NCoR/SMRT complexes (NCoR, SMRT, TBL1, TBLR1). This approach confirmed the interaction of Chk2 with SMRT, TBL1 and TBLR1, but not with NCoR (Figure S1C). When the same experiment was performed using flag-tagged Chk1 for comparison, no interaction was detected.

To initially explore if Chk2 had an effect on transcription, an array of luciferase expression vectors containing binding sites for different transcription factors (AP1, NFκB and RAR), which use NCoR/SMRT as co-repressors, were used to perform standard reporter assays. Knock-down of Chk2 in U2OS cells potentiated the transcriptional response to AP1 and RAR-driven reporters (Figure S1D), suggesting a repressive function for Chk2.

### SMRT, but not NCoR, significantly affects DNA damage-induced transcription

Because Chk2 is a major regulator of DNA damage checkpoint response, we sought to determine potential roles of the NCoR/SMRT co-repressor complex in the transcriptional response to DNA damage. Osteosarcoma cells U2OS were chosen because they have a functional p53 and represent a good model for studying DNA damage checkpoint. A time-course experiment in U2OS cells showed that the genotoxic drug cisplatin (CDDP) at the concentration of 100 µM caused phosphorylation of Chk2 on T68 starting at 4 h and reaching a peak at 6 h (Figure S2). The peak of Chk2 activation was followed by caspase activation reaching a plateau at 8 h (Figure S2) Caspase activation was evaluated through an antibody that specifically recognizes the PARP fragment after the cleavage by caspase 3 on Asp214, while not interacting with the full-length protein or with other PARP degradation fragments (Figure S2).

**Figure 1:**
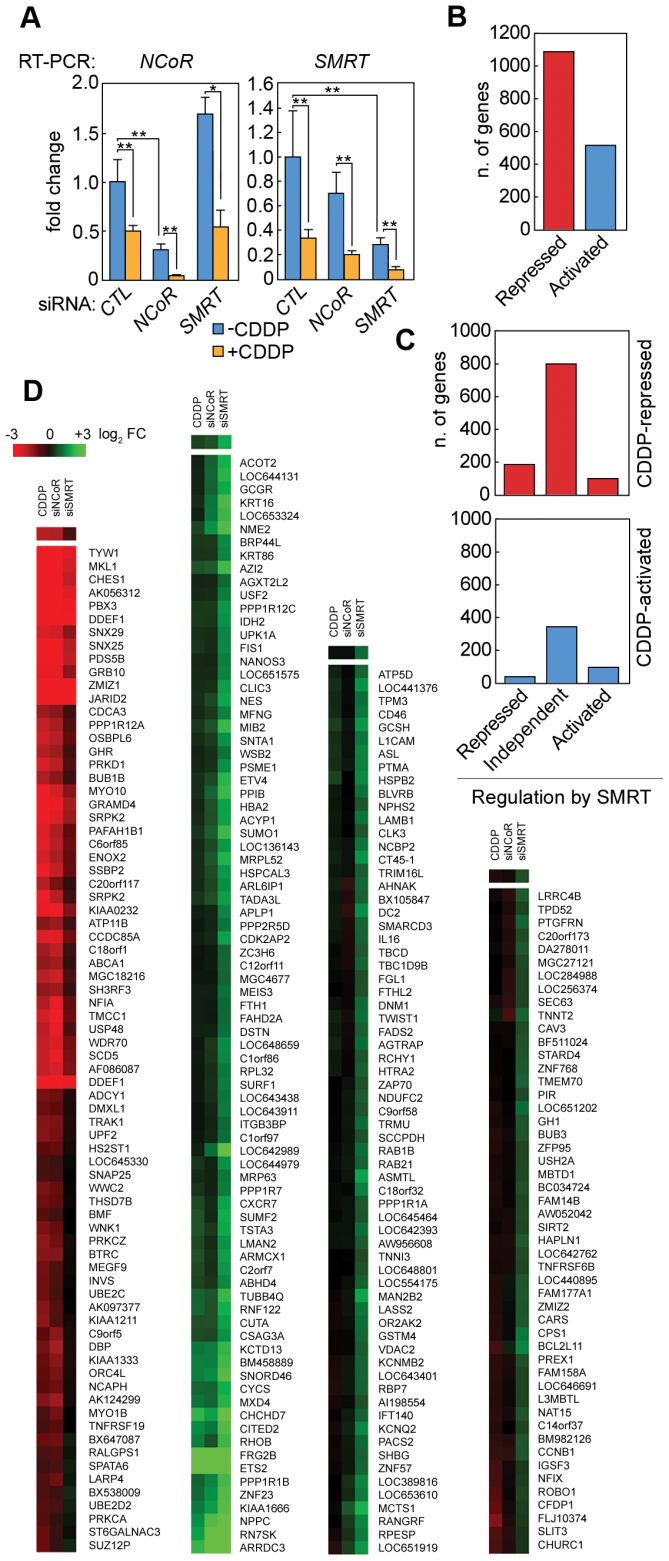
SMRT, but not NCoR, affects CDDP-induced transcriptional program. A) U2OS cells were transfected with scramble siRNA or with siRNAs for *NCoR* or *SMRT*, and then treated with 100 µM CDDP for 6 h, followed by RNA extraction and RT-qPCR with primers specific for *NCoR* (left panel) or *SMRT* (right panel). Results from three independent experiments, each with three technical replicates, were analyzed by the ΔΔCt method, using 18S as a normalizer. (Student's T-test; one star: p-value≤0.05, two stars: p-value≤0.01, three stars: p-value≤0.001). B–D) RNAs from two of the biological replicates described in A were subjected to labeling and hybridization on Illumina BeadChip arrays. B) The histogram indicates the number of genes that are either activated or repressed by CDDP, using a log_2_ fold change cut-off of 0.585. C) The two histograms report the number of genes whose regulation by CDDP is affected by *SMRT* knock-down, grouped by CDDP repression (upper panel) or activation (lower panel). D) Genes regulated by CDDP differently in the *SMRT* knock-down compared to the *NCoR* knock-down. The criteria for selection of SMRT-specific genes were the following: false discovery rate ≥0.2; log_2_ fold change (CDDP/vehicle) in scramble siRNA cells ≥|0.32|; difference between log_2_ fold changes in the *SMRT* knock-down and the scramble siRNA [(siSMRT+CDDP/siSMRT+vehicle) – (siCtl+CDDP/siCtl+vehicle)] ≥0.32; difference between log_2_ fold changes in the *SMRT* knock-down and the *NCoR* knock-down [(siSMRT+CDDP/siSMRT+vehicle) – (siNCoR+CDDP/siNCoR+vehicle)] ≥0.32. Genes were grouped with the “Cluster” software (using the “Average linkage clustering” function) and the heat maps were created with the “TreeView” software.

To study the role of NCoR and SMRT in CDDP-induced gene transcription, U2OS cells were transfected with siRNAs against *SMRT* or *NCoR*, and 48 hours later treated with 100 µM CDDP for 6 h to induce damage and activate Chk2. RNA was extracted, labeled and hybridized on Illumina BeadChip microarrays. To control the efficiency of knock-down, RT-qPCR was performed with primers specific for *NCoR* or *SMRT*. As shown in Figure 1A, the siRNAs against *NCoR* and *SMRT* caused a reduction in the respective mRNA levels of 71% and 65%. The microarray profiling showed that treatment with CDDP induced a massive wave of gene repression, with negatively-regulated genes (1074) being twice as frequent as positively-regulated (507) ones (Figure 1B and Table S1), suggesting that transcriptional repression is an important aspect of the response to CDDP-induced DNA damage. Gene Ontology analysis of CDDP-regulated genes showed enrichment, among the most significant biological processes, of negative regulation of transcription, cell cycle, apoptosis, and cell death (Table 1). The reduction of NCoR levels did not significantly affect the CDDP-induced transcriptional program, with only 36 genes being significantly regulated by CDDP differently in the *NCoR* knock-down compared to the scramble siRNA-transfected cells (FDR ≥0.2, Table S2). Conversely, knock-down of *SMRT* resulted in significant changes in the transcriptional program triggered by CDDP (Figure 1C and Table S3). Among the CDDP-repressed genes, 186 (16%) were no longer repressed or were significantly less repressed with *SMRT* knocked-down (Figure 1C, upper panel and Class 1 in Table S3), while 99 genes (9%) where repressed more intensely in the absence of SMRT. Among the CDDP-activated genes, 37 (7.8%) were activated more intensely in the *SMRT* knock-down cells (Figure 1C, lower panel), indicating that this co-repressor limits the activation of these genes after CDDP treatment, while 94 (19.9%) were not activated or activated less intensely when *SMRT* was knocked down. Moreover, 17% of the genes activated by treatment with CDDP were also activated by SMRT knock-down in the absence of treatment (Class 2 in Table S3), suggesting a basal repression by SMRT, which is removed by treatment with CDDP. Figure 1D reports a heat map of a selected group of genes regulated by CDDP differently in the *SMRT* knock-down compared to the *NCoR* knock-down, showing how the profile in the *NCoR* siRNA resembled the profile in the scramble siRNA-transfected cells, while the *SMRT* siRNA stood out for both activation and repression of genes. Interestingly, in the group of genes that were differentially regulated by CDDP in the cells where *SMRT* was knocked down compared to scramble siRNA-transfected cells, some of the most enriched GO terms were cell death and apoptosis, along with protein amino acid phosphorylation (Table 2).

**Table 1:** Most enriched Gene Ontology (GO) terms in CDDP-regulated genes.

GO ID	Biological Process	p
45941	positive regulation of transcription	2.77E-14
6468	protein amino acid phosphorylation	5.51E-13
122	negative regulation of transcription from RNA polymerase II promoter	4.28E-12
30154	cell differentiation	5.98E-12
51093	negative regulation of developmental process	1.65E-11
7049	cell cycle	3.51E-11
7167	enzyme linked receptor protein signaling pathway	3.65E-11
42127	regulation of cell proliferation	5.74E-11
1568	blood vessel development	1.07E-09
43066	negative regulation of apoptosis	1.31E-09
6915	apoptosis	3.47E-09
51094	positive regulation of developmental process	3.67E-09
8219	cell death	3.71E-09

**Table 2:** Most enriched Gene Ontology (GO) terms in genes whose regulation by CDDP is affected by SMRT.

GO ID	Biological Process	p
6468	protein amino acid phosphorylation	7.33E-13
8219	cell death	8.35E-13
7049	cell cycle	5.34E-12
6915	apoptosis	7.86E-12
12501	programmed cell death	1.37E-11
33554	cellular response to stress	3.71E-10
10558	negative regulation of macromolecule biosynthetic process	1.00E-09
6357	regulation of transcription from RNA polymerase II promoter	1.13E-09
10629	negative regulation of gene expression	1.43E-09
9966	regulation of signal transduction	1.62E-09
51094	positive regulation of developmental process	1.79E-09
16481	negative regulation of transcription	1.92E-09
8283	cell proliferation	2.41E-09
43933	macromolecular complex subunit organization	3.40E-09
19941	modification-dependent protein catabolic process	3.57E-09
10926	anatomical structure formation	5.46E-09
44085	cellular component biogenesis	5.86E-09

### SMRT protects against apoptosis through repression of pro-apoptotic genes

**Figure 2:**
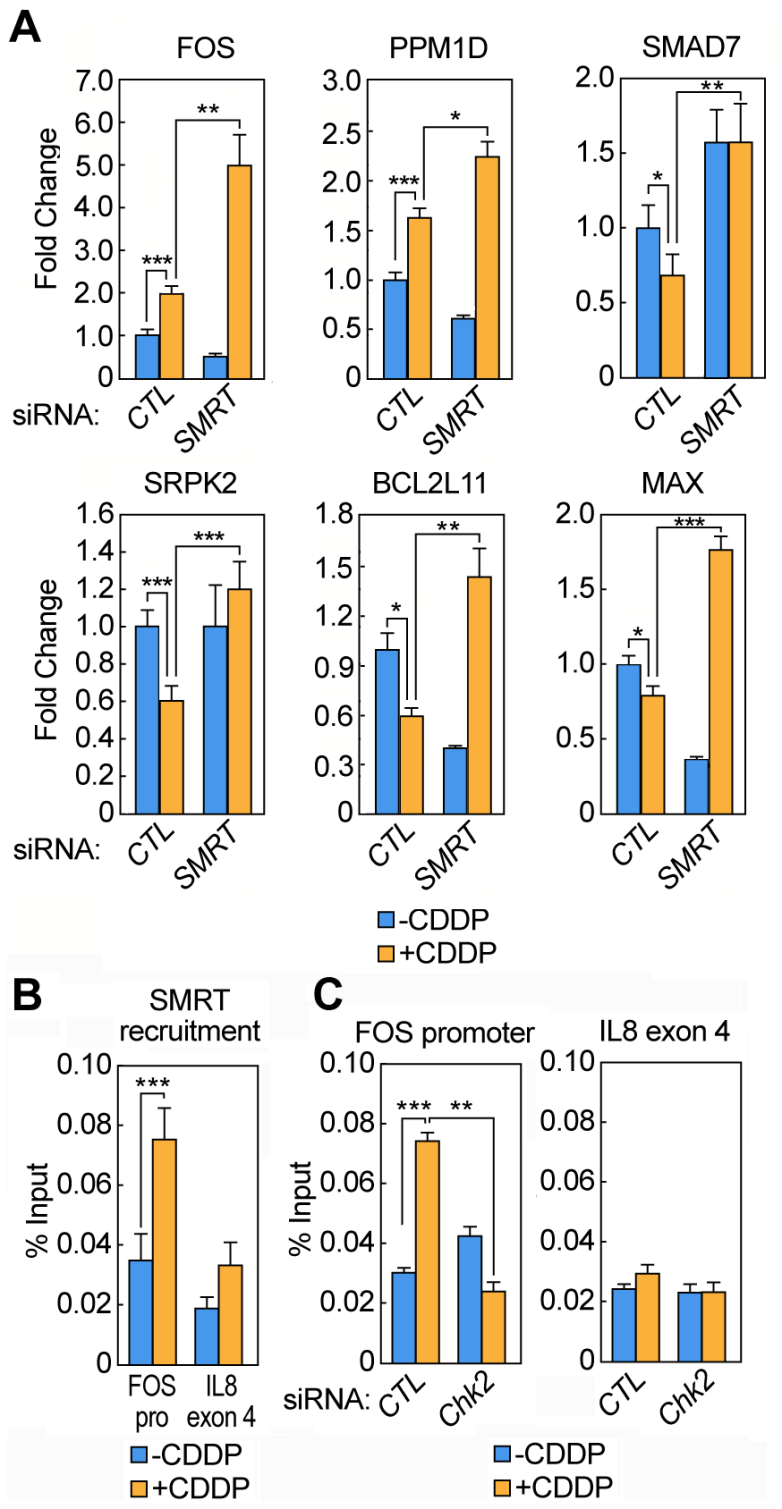
SMRT represses a group of pro-apoptotic genes. A) RT-qPCR was performed on RNA extracted from U2OS cells after treatment for 6 h with 100 µM CDDP, with or without siRNA against *SMRT*. Results from three independent experiments, each with three technical replicates, were analyzed by the ΔΔCt method, using 18S as a normalizer. B) Chromatin immunoprecipitation with an antibody specific for SMRT after treatment of 293 cells with 100 µM CDDP for 2 h. The qPCR was performed on the immunoprecipitated DNA, and percentage of the input was calculated by comparing the sample Ct with a curve made of four serial 1/5 dilutions of 1% input. The results are the average of three independent experiments, each one with three technical replicates. C) Same as in B, but with transfection of either scramble siRNA or siRNA against *Chk2* and qPCR with specific primers for *FOS* promoter (left panel) or *IL8* exon 4 (right panel). The results are the average of three independent experiments, each one with three technical replicates. (Student's T-test performed for all experiments in the figure; one star: p-value≤0.05, two stars: p-value≤0.01, three stars: p-value≤0.001).

Because apoptosis was among the most enriched Gene Ontology (GO) terms in SMRT-dependent genes, we selected a group of pro-apoptotic genes regulated by CDDP (*FOS, PPM1D, SMAD7, SRPK2, BCL2L11, MAX*), and performed RT-qPCR after transfection of the cells with siRNA against *SMRT* and subsequent treatment with CDDP. As shown in Figure 2A, SMRT limited CDDP-dependent activation of *FOS* and *PPM1D* and exhibited a repressive function on *SMAD7, SRPK2, BCL2L11* and *MAX*, as their inhibition by CDDP was lost in the cells transfected with *SMRT* siRNA. Because AP1 is a transcription factor implicated in induction of apoptosis, itself utilizing the NCoR/SMRT complex for repression of target genes, the regulation of *c-FOS*, a major component of the AP1 family, was selected for further investigation. Chromatin immunoprecipitation of SMRT following CDDP treatment revealed increased recruitment of SMRT on the *c-FOS* promoter after DNA damage (Figure 2B), suggesting direct regulation of *c-FOS* by SMRT. Knock-down of *Chk2* was able to abrogate the occupancy of SMRT on the *c-FOS* promoter, supporting the requirement for Chk2 in DNA damage-dependent co-repressor recruitment (Figure 2C).

**Figure 3:**
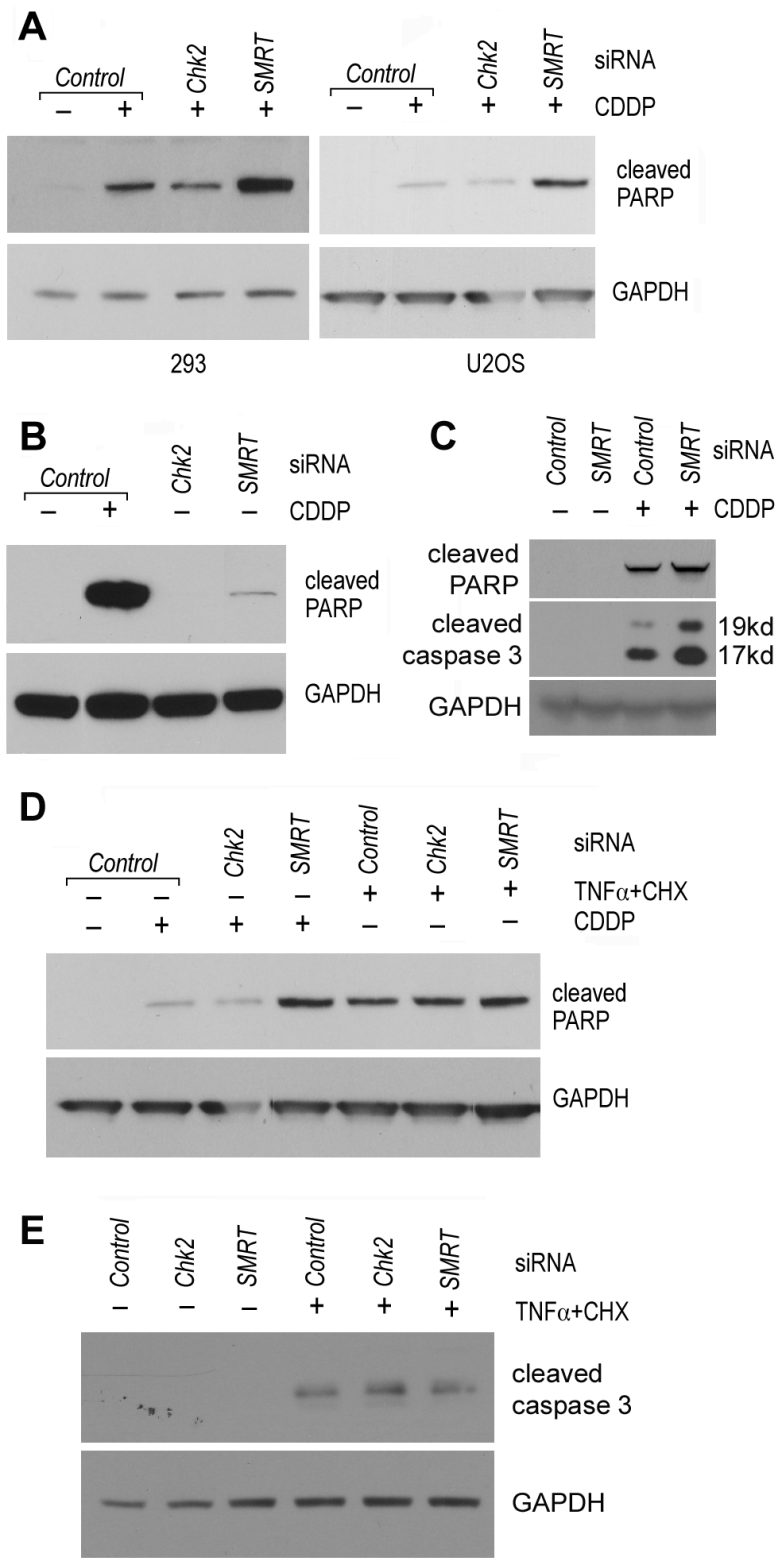
SMRT has a protective action against DNA damage-induced caspase activation. A–C) 293 and U2OS cells were transfected with the indicated siRNAs and treated with either DMSO or 100 µM CDDP for 8 h, followed by Western blot analysis on whole cell extracts with the indicated antibodies. D–E) U2OS cells were transfected with the indicated siRNAs and treated with either 100 µM CDDP or 20 ng/µl TNF-α + 5 µg/ml cycloheximide for 6 h. All shown data is representative of at least three independent experiments. GAPDH is used as a loading control.

In order to investigate the biological consequences of the Chk2-SMRT regulatory events, U2OS and 293 cells were transfected with siRNAs against *Chk2* or *SMRT*, followed by treatment with toxic doses of CDDP and Western blot analysis of the caspase 3-dependent cleavage of PARP1. As shown in Figure 3A, while the siRNAs against *Chk2* had little effect on the activation of caspase 3, knock-down of *SMRT* increased PARP cleavage in CDDP-treated cells. The knock-down of *SMRT* caused a very slight increase in PARP cleavage also in non-treated cells, only visible after very long exposure (Figure 3B). To confirm activation of caspase 3 by *SMRT* siRNA, a Western blot was performed on U2OS protein extracts with an antibody which specifically recognized the 17-kDa and 19-kDa cleavage products of caspase 3, showing increased caspase 3 cleavage when SMRT was knocked-down (Figure 3C). This effect was not detected in cells transfected with the same siRNAs, but treated with TNF-α in combination with cycloheximide, which has been shown to activate the extrinsic apoptotic pathway by the TNF receptor [58] (Figure 3D–E).

### SMRT represses the phosphatase Wip1, influencing the dynamics of Chk2 activation

**Figure 4:**
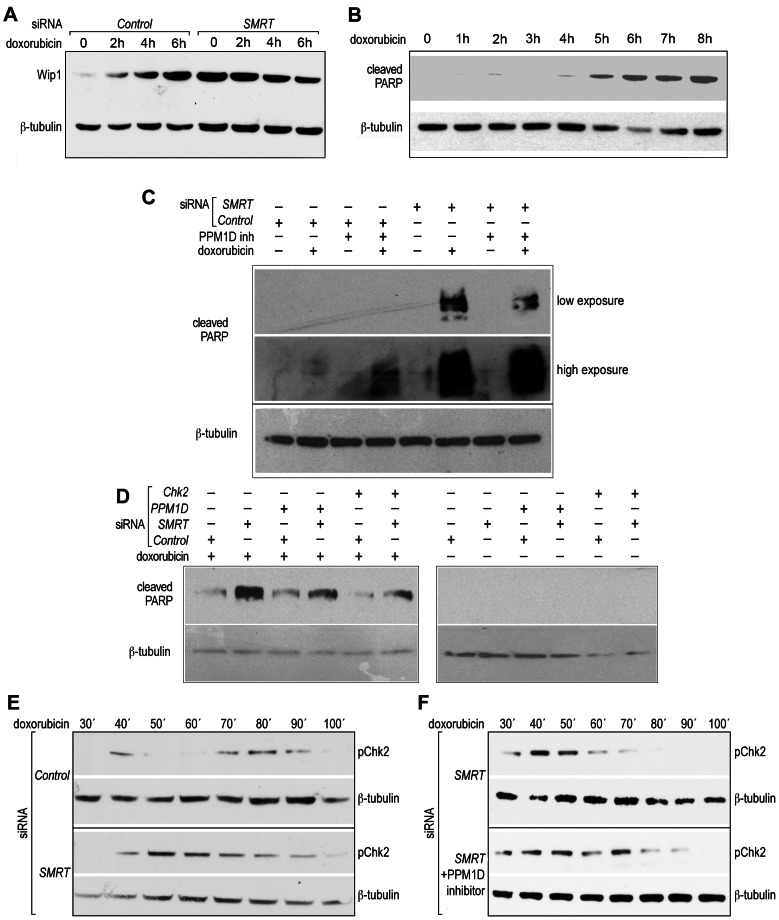
Wip1 and Chk2 are required for activation of caspase by SMRT knock-down after DNA damage. A) U2OS cells were transfected with scramble siRNA (Ctl) or siRNA against *SMRT* and then treated with 5 µM doxorubicin for the indicated time points, followed by protein extraction and Western blot with the indicated antibodies. B) U2OS cells were treated with 5 µM doxorubicin for the indicated time points and protein extracts were used for Western blot. C) U2OS cells were transfected either with scramble siRNA or with siRNA against *SMRT* and then treated for 6 h with 5 µM doxorubicin with or without co-treatment with 50 µM PPM1D inhibitor, and the protein extracts were used for Western blot with antibodies specific for cleaved PARP1 and tubulin. D) U2OS cells were transfected with the indicated siRNAs and then treated with for 6 h with 5 µM doxorubicin; the protein extracts were used for Western blot with antibodies specific for cleaved PARP1 and tubulin. E) U2OS cells were treated with 5 µM doxorubicin at 10′ intervals for 2 h, and protein extracts were analyzed by Western blot with phospho-Chk2 (T68) or tubulin antibodies, both in control cells and in the *SMRT* knock-down. F) Cells were transfected with siRNA against *SMRT* and treated with doxorubicin for the indicated time points, with (lower panel) or without (upper panel) additional treatment with PPM1D inhibitor. All shown data is representative of at least three independent experiments.

Interestingly, SMRT was required for CDDP-induced repression of the Wip1 phosphatase (*PPM1D* gene, Figure 2A), a major down-regulator of the Chk2 pathway, as well as of other phosphorylated proteins such as p53 and p38 [55]. To confirm the repressive action of SMRT on *PPM1D*, U2OS cells were transfected with siRNA against *SMRT* and treated with another genotoxic drug, doxorubicin, which inhibits topoisomerase II and causes double strand breaks. As expected, Wip1 protein levels were augmented by doxorubicin treatment and increased by siRNA against SMRT, even in the absence of DNA damage, indicating basal repression of *PPM1D* by SMRT (Figure 4A).

We next investigated the role of *PPM1D* repression in the initial caspase activation induced by *SMRT* knock-down in the presence of DNA damage. We first treated the cells with doxorubicin for different time points, and detected cleavage of PARP starting between 5 h and 6 h of treatment (Figure 4B). While knock-down of *SMRT* caused an increase in doxorubicin-induced caspase 3 activation, co-treatment with a chemical inhibitor of Wip1 attenuated this phenotype (Figure 4C). Consistently, co-transfection of siRNA against *PPM1D* reduced caspase activation when compared to actions of *SMRT* siRNA alone (Figure 4D, compare lane 4 and 2).

Wip1 has many substrates, all involved in the regulation of apoptosis in response to DNA damage or cellular stresses. To determine whether Chk2 is required for the activation of caspase that occurs when *SMRT* is down-regulated, we co-transfected U2OS cells with siRNAs for *SMRT* and *Chk2*. Indeed, co-transfection with *Chk2* siRNA attenuated the caspase activation caused by *SMRT* knock-down, after treatment with doxorubicin (Figure 4D, compare lanes 6 and 2).

Because the Wip1-dependent feedback has been reported to cause oscillatory behavior in Chk2 activation [24], [59], and because in genetic oscillators the intensity of the feedback loop dictates the oscillatory behavior of the system [60]–[62], we sought to examine the possibility that the SMRT-dependent repression of *Wip1* might be responsible for influencing the dynamics of Chk2 activation after DNA damage. U2OS cells were treated with doxorubicin at 10′ intervals for 2 hours, comparing cells transfected with scramble siRNA or siRNA against *SMRT*. In control conditions, an oscillatory pattern of Chk2 phosphorylation was observed, with two discrete peaks at 40′ and 80′. SiRNA against *SMRT* altered this pattern, resulting in only one, more persistent phospho-Chk2 peak (Figure 4E). Interestingly, when the cells transfected with the *SMRT* siRNA were also treated with a Wip1 inhibitor, the single peak of Chk2 activation (Figure 4F, upper panel) was replaced by two peaks of activation after doxorubicin treatment, one at 40′–50′ and one at 70′ (Figure 4F, lower panel). Even if the peaks are not perfectly overlapping with the original oscillation observed in the wild type cells, this result suggests that the lack of inhibition of Wip1 might play a role in the altered dynamics observed in the *SMRT* knock-down.

### SMRT is required for proper repair of DNA damage

**Figure 5:**
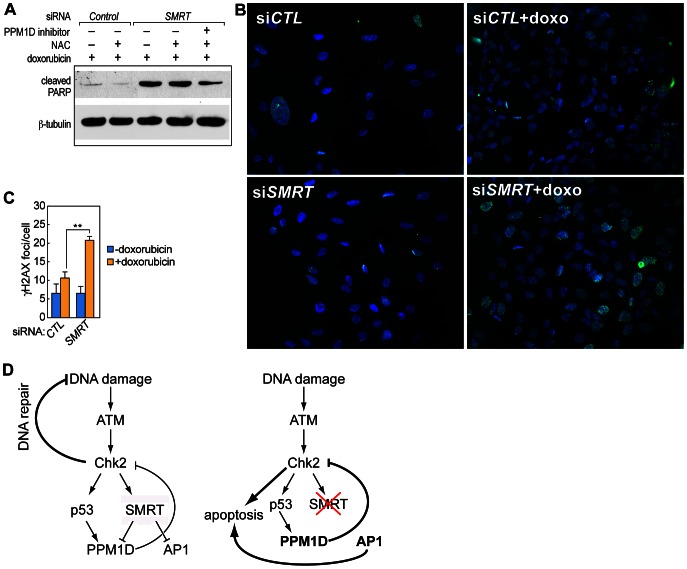
SMRT is required for monitoring the DNA repair process. A) U2OS cells were transfected with scramble siRNA (Ctl) or siRNA against *SMRT* and then treated with 5 µM doxorubicin for 6 h, with or without co-treatment with the antioxidant 10 µM N-Acetyl-cysteine (NAC). The data presented is representative of at least three independent experiments. B–C) U2OS cells were transfected with scramble siRNA or siRNA against *SMRT* and treated with 5 µM doxorubicin for 1 h, followed by fixation and immuno-staining with γH2AX antibody. Representative pictures are reported in B. Five images for each experimental point were taken for each experiment. γH2AX foci from three independent experiments were quantified by ImageJ software, and the average number of foci per cell was plotted in C. (Student's T-test; one star: p-value≤0.05, two stars: p-value≤0.01, three stars: p-value≤0.001). D) Model of modifications in the ATM-Chk2-PPM1D system when SMRT is removed.

It has been argued that the cycles of oscillation in checkpoint proteins allow cells to monitor DNA repair and re-activate the checkpoint response if the damage is still present at the end of each cycle [24], [63]. To assess the applicability of this model to Chk2, we treated U2OS cells with doxorubicin with or without co-treatment with the antioxidant N-Acetyl-Cysteine (NAC), which reduces the amount of DNA damage by scavenging free radicals. As expected, treatment with the antioxidant reduced caspase activation in response to doxorubicin. However, if the cells were transfected with siRNA specific for *SMRT*, the ability of the antioxidant to rescue the DNA damage-induced apoptosis was lost (Figure 5A), suggesting that SMRT is involved in monitoring repair and shutting down the response if the DNA damage is repaired. Interestingly, co-treatment with the Wip1 inhibitor partially restored the ability of the *SMRT*-deficient cells to block apoptosis in the presence of NAC (Figure 5A, lane 5), suggesting a role of Wip1 de-repression in the observed phenomenon.

Finally, we argued that if SMRT is required for properly sensing and responding to DNA damage, then *SMRT* knock-down should affect the ability of the cells to repair DNA damage. As expected, when *SMRT* was down-regulated by siRNA, the number of γH2AX foci, a marker of active repair of DNA double strand breaks, was doubled (Figure 5B–C).

## Discussion

The decision between life and death of a cell is a very important one, with relevant implications for cancer therapy. Here we present a role of the transcriptional co-repressor SMRT in delaying the induction of apoptosis after DNA damage. We suggest that Checkpoint kinase 2, activated by DNA damage, induces recruitment of SMRT on the promoters of some pro-apoptotic genes, thus delaying activation of caspase and allowing DNA repair (Figure 5D, left panel). When SMRT is removed from the cells, pro-apoptotic genes are de-repressed and cells activate the caspase pathway (Figure 5D, right panel). Our data showed that SMRT is down-regulated both at the RNA level (Figure 1A) and at the protein level (data not shown) after treatment with apoptotic doses of CDDP.

The RNA profiling results suggest that SMRT plays a significant role in gene repression after DNA damage. However, SMRT actually exhibited an activating action on some CDDP-regulated genes, possibly due to secondary effects. The genes that we confirmed by RT-qPCR to be inhibited by SMRT are all important regulators of apoptosis, belonging to different pathways. *BCL2L11* (or *Bim*) is a member of the “BH3-only” family of apoptosis facilitators; members of this family bind and inactivate pro-survival Bcl2-like proteins, thus allowing permeabilization of mitochondrial membrane by Bax/Bak channels [64]. *SRPK2* is a protein Serine-Arginine kinase that, after DNA damage, migrates to the nucleus and induces apoptosis through only partially defined mechanism [65]. *MAX* forms a functional dimer with *MYC* and binds the promoter of target genes thus inducing cell proliferation as well as apoptosis [66], [67]. Of particular interest, among the pro-apoptotic genes repressed by SMRT there are some components or regulators of two major pathways involved in the regulation of apoptosis: AP1 and NFκB. *FOS* is a major transcription factor of the AP1 family [68], and has been associated with induction of apoptosis after different cellular stresses, including DNA damage [31], [32]. *SMAD7* is an inhibitory transcription factor activated by TGF-β signaling, required for repression of anti-apoptotic NFκB target genes [69]. The SMRT complex is already known to be required for gene repression by AP1 [47] and NFκB [46]. In our experiments, SMRT exerts a basal repressive function on NFκB genes, as its removal from the cells by siRNA induces up-regulation of NFκB targets such as *PPM1D* (Figure 2A) as well as *IL-8* and *TNF-α* (data not shown). In contrast, active recruitment of SMRT on the promoter of AP1 targets such as *c-FOS* occurs only after DNA damage.

As expected, the *SMRT* knock-down sensitizes cells to DNA damaging agents such as CDDP and doxorubicin, increasing drug-induced caspase activation. An apparently inconsistent finding is that the *Chk2* knock-down does not have the same effect. Although we were not able to demonstrate direct phosphorylation of SMRT by Chk2, our data suggest that phosphorylation of SMRT occurs after treatment with CDDP, which is reduced, but not entirely abolished, by *Chk2* siRNA (data not shown). Therefore, we are tempted to suggest that SMRT might be phosphorylated by different kinases after DNA damage, such as Chk1, p38, and JNK, which can complement the absence of Chk2.

Of particular interest to us is the SMRT-dependent repression of the protein phosphatase Wip1 or PPM1D, a down-regulator of the Chk2 pathway. De-repression of *Wip1* by *SMRT* knock-down is associated with increased caspase activation. It is tempting to speculate about potential explanations of these observations. One hypothesis is that Wip1 de-repression changes the kinetics of Chk2 activation. Chk2 has been reported to be activated in cycles [24]. It has been shown that the frequency of oscillations of a negative feedback system is dictated by the balance between the activating stimulus and the negative feedback [60], [62]. Therefore, Chk2 oscillations might change in frequency as the system evolves: initially, when Wip1 levels are low, one observes high-frequency oscillations (40′ period); however, as Wip1 levels increase due to p53-dependent transcriptional activation (mimicked by *SMRT* knock-down), one might expect lower frequency oscillations and, therefore, more persistent phosphorylation of Chk2 and activation of caspases. While more detailed kinetic analysis of Chk2 activation would be required to fully characterize the system's evolution during time, the idea of frequency modulation as a strategy to induce different cellular outcomes is a possible explanation.

Indeed, the idea of alteration in activation dynamics as a regulatory strategy has been previously proposed. The ATM-Chk2-p53 pathway has been shown to be activated in cycles after IR treatment [24]; it has been suggested that the cycles of p53 activation are required to sense the intensity of the DNA damage [59] and to modulate the response according to the severity of the damage [70]. Moreover, the kinetics of activation of JNK-1 has been proposed to dictate the cellular outcome, with transient activation being pro-inflammatory and pro-survival, while sustained activation inducing cell death [71].

The mechanism of the inhibition of *PPM1D* gene by SMRT is not clear. *PPM1D* has been reported to be regulated by NFκB [72], which uses SMRT as a co-repressor. However, we were not able to detect any SMRT protein on the NFκB binding site in the *PPM1D* promoter (data not shown). It is possible that the effect on Wip1 is indirect, mediated by increased autocrine secretion of TNF-α by the *SMRT* knock-down cells. Indeed, treatment of the cells with exogenous TNF-α has an effect on caspase activation similar to that of siRNA for *SMRT*: increased doxorubicin-induced apoptosis, which can be rescued by siRNA for Wip1 (data not shown).

Finally, Wip1 regulates many different kinases [55] and, therefore, its regulation has most likely wider consequences than only those dependent on Chk2 oscillation. We speculate that oscillatory behavior occurs simultaneously in many different systems and for many different regulatory proteins, wherever a negative feedback loop is present, and the final “decision” on cell fate depends on the balance between the signals sent by each of the regulatory checkpoint proteins.

## Materials and Methods

### Antibodies, siRNAs, chemicals and cell lines

Antibodies specific for NCoR were generated in our laboratory as previously described (50). All the other antibodies were commercial: Flag (SIGMA), cleaved PARP (human-specific), cleaved caspases 3 (Asp175) and phospho-Chk2 (T68) (Cell Signaling Technology), SMRT (Abcam for Western blot, Affinity Bioreagents for chromatin immunoprecipitation), GAPDH (Santa Cruz Biotechnology), β-tubulin (SIGMA), γH2AX (Upstate, mouse monoclonal). The following chemicals were used: TNF-α, PPM1D inhibitor (Calbiochem), TPA, 9cis-RA, cisplatin, doxorubicin, cycloheximide and N-acetyl-cysteine (SIGMA).

SiRNAs specific for Chk2, NCoR, SMRT and PPM1D were purchased from Invitrogen, and a mix of three independent siRNAs was used for each gene.

All cell lines (U2OS, HeLa, 293) were purchased from ATCC.

### Cell culture

All cells were grown in Dulbecco Modified Eagle Medium (GIBCO) with low glucose, supplemented with 10% FBS and 50 µg/ml Gentamycin. For transfection, Lipofectamine 2000-based liposomes were incubated in serum-free medium (5 ml/plate) and Optimem medium (3 ml/plate) for 6 hours, followed by incubation in complete medium for 2 days before the appropriate treatments. Cells were incubated for 2 days in serum-free DMEM medium before treatments with 9cisRA, TNF-α or TPA.

### Reverse Transcription-qPCR

Total RNA was extracted with RNEasy kit (QIAGEN) according to the manufacturer's recommendations, and 1 µg of RNA was used for RT reaction by SuperScript III Reverse Transcriptase kit (Invitrogen); cDNA samples were diluted 1∶20 for test genes and 1∶200,000 for the reference gene (18S), and 4 µl per reaction were used for qPCR, using Brilliant III SYBR Green master mix (Agilent). Results from three technical replicates were analyzed by the ΔΔCt method, using 18S as a normalizer. Briefly, statistical significance was calculated by normalizing the values of three independent biological replicates for the respective 18S values, eliminating the outliers and averaging the ΔCts; the p-value was calculated by Student's T-test.

### Chromatin Immunoprecipitation

After the appropriate transfections and treatments, HEK293 cells were fixed by incubation for 10′ with 2% formaldehyde and then in 0.125 M Glycine for 15′. Cells were harvested in PBS, the pellet was washed sequentially in ChIP Buffer I (0.25% Triton X-100, 10 mM EDTA, 0.5 mM EGTA, 10 mM HEPES pH 6.5) and ChIP Buffer II (200 mM NaCl, 1 mM EDTA, 0.5 mM EGTA, 10 mM HEPES pH6.5) and then incubated for 1 h in Lysis Buffer (1% SDS, 10 mM EDTA, 50 mM Tris-HCl pH 8.1, 1X Complete protease inhibitor mix [Roche]). Chromatin was sonicated by BioRuptor (Diagenode, 8 pulses, 5′ each on High setting, 50% time pulses), diluted 1/10 in Dilution buffer (1% Triton X-100, 2 mM EDTA, 150 mM NaCl, 20 mM Tris-HCl pH 8.0, protease inhibitor mix) and immunoprecipitated with 5 µl NCoR or SMRT antibodies over night, followed by incubation for 2 h with protein A sepharose (Invitrogen, 50 µl/sample of 50% slurry) or protein A Dynabeads (Invitrogen, 15 µl/sample of 30 mg/ml mix). The immunoprecipitate was washed sequentially in TSE-I (0.1% SDS, 1% Triton X-100, 2 mM EDTA, 20 mM Tris-HCl pH8.0), TSE-II (0.1% SDS, 1% Triton X-100, 2 mM EDTA, 20 mM Tris-HCl pH 8.0, 500 mM NaCl), Buffer III (0.25 M LiCl, 1% NP-40, 1% deoxycholate, 1 mM EDTA, 10 mM Tris-HCl pH 8.0) and twice in TE Buffer, then resuspended in Elution Buffer (1% SDS, 0.1 M NaHCO_3_) and de-crosslinked at 65°C for 6 hrs. DNA was extracted through QIAquick Spin columns (QIAGEN), diluted 1∶4 and 4 µl were used for qPCR, using technical triplicates. Percentage of the input was calculated by performing four serial 1/5 dilutions of 1% input. All qPCR reactions were performed as described for RT-qPCR. The results shown derive from averaging three independent experiments; the statistical significance was calculated by Student's T-test.

### Microarrays

U2OS cells were transfected with siRNAs for *NCoR* or *SMRT*, incubated in complete medium for 2 days, and then treated with 100 µM CDDP for 6 hrs, followed by RNA extraction, labeling with Illumina TotalPrep RNA Amplification Kit (Ambion) and hybridization on Illumina Expression Beadchips (HumanRef 8.0), following the manufacturer's recommendations. Data was analyzed as described in Methods S1. The data was uploaded on the GEO database (ID number: GSE34226).

### Cell extracts and fractionation

For whole cell extracts, Cells were washed in cold PBS, harvested and resuspended in IPH buffer (50 mM Tris-HCl pH 8.0, 150 mM NaCl, 5 mM EDTA, 0.5% NP-40, 1 mM PMSF, 50 mM NaF, 2 mM Na_2_VO_3_, 1X Complete protease inhibitor mix [Roche] and 1 mM glycerol phosphate), incubated for 20′ on ice and subsequently centrifuged for 10′ at 13,000 rpm. The supernatant (whole cell extract was used for Western blot analysis.

### Immunofluorescence

U2OS cells were plated at 80% confluence in 8-well plates, transfected as indicated with Lipofectamine 2000 (Invitrogen) and then treated with 5 µM doxorubicin for 1 h. Cells were fixed in 2% paraformaldehyde for 10′ at room temperature and then washed in PBS 4 times (20′ each), followed by permeabilization in 0.1% NP-40 in PBS (30′ at room temperature) and incubation in PGBA-Super blocking solution (0.1% gelatin, 10% BSA, 0.01% sodium azide, 1% normal goat serum in PBS) for 30′ at room temperature. Primary antibody anti-γH2AX was diluted 1∶200 in PGBA solution (0.1% gelatin, 1% BSA, 0.01% sodium azide in PBS) and added to the cells over-night. Cells were washed 4 times in PBS and then incubated for 1 h with secondary antibody anti-mouse (1∶300 in PGBA) conjugated with Alexa-488 (Invitrogen). Cells were washed 3 times in PBS and then analyzed at the fluorescence microscope at 20X magnification. Five pictures were taken for each experimental point. γH2AX foci were counted with ImageJ, using the FociPicker3D application, followed by counting the number of cells for each picture with the ‘Analyze Particles’ function, and the number was used to calculate the average number of foci per cell. Data from three independent experiments were used to calculate statistical significance, with Student's T-test.

## Supporting Information

Figure S1 **Purification of proteins interacting with the FHA of Chk2.** A) A PATH-tagged FHA domain of Chk2 was purified from bacteria and incubated with cellular extracts from HeLa cells. As a control, a mutant lacking the ability to bind target phosphopeptides was used. Purified proteins were run on polyacrylamide gels and visualized with silver stain. B) Proteins identified to specifically interact with the FHA domain of CHK2. C) U2OS cells were transfected with Flag-tagged *Chk1* or *Chk2* expression vectors, and protein extracts were immunoprecipitated with anti-Flag antibody, followed by SDS-PAGE and Western Blot analysis. D) U2OS cells were transfected with the indicated reporters with or without siRNA against *Chk2*, incubated for 2 days in serum-free medium and then treated with 10 ng/µl TPA, 20 ng/µl TNF-α or 5×10^−8^ M 9cisRA, as indicated. Statistical significance was calculated on the ratio between luciferase luminescence and Renilla luciferase from three independent experiments, each one including four technical replicates, by Student's T-test. (One star: p-value≤0.05, two stars: p-value≤0.01, three stars: p-value≤0.001).
(TIF)

Figure S2 **Time-course of cisplatin (CDDP) treatment.** U2OS cells were treated with 100 µM CDDP for the indicated time points and the whole cell extracts were used for Western blot with specific antibodies against phospho-Chk2 (T68), cleaved PARP (Asp214), or β-tubulin.
(TIF)

Table S1 **Genes regulated by cisplatin.** The table reports the total number of genes resulting regulated by CDDP in the microarrays experiment, ranked by significance as described in Methods S1.
(XLS)

Table S2 **Genes whose regulation by cisplatin is affected by NCoR.** The table reports the total list of genes whose regulation by CDDP in the microarray experiments was different in the cells transfected with *NCoR* siRNA compared to the cells transfected with scramble control siRNA.
(XLS)

Table S3 **Genes whose regulation by cisplatin is affected by SMRT.** The table reports the total list of genes whose regulation by CDDP in the microarray experiments was different in the cells transfected with *SMRT* siRNA compared to the cells transfected with scramble control siRNA.
(XLS)

Methods S1 **Methods for the data included in Supporting Figures and detailed descriptions of microarray data analysis, plasmids and qPCR primers are included.**
(DOC)
