# Supplementary figures and images for: The Co-Repressor SMRT Delays DNA Damage-Induced Caspase Activation by Repressing Pro-Apoptotic Genes and Modulating the Dynamics of Checkpoint Kinase 2 Activation
Source: PLoS One. 2013 May 17;8(5):e59986. doi: 10.1371/journal.pone.0059986 (PMC3656868; doi:10.1371/journal.pone.0059986)

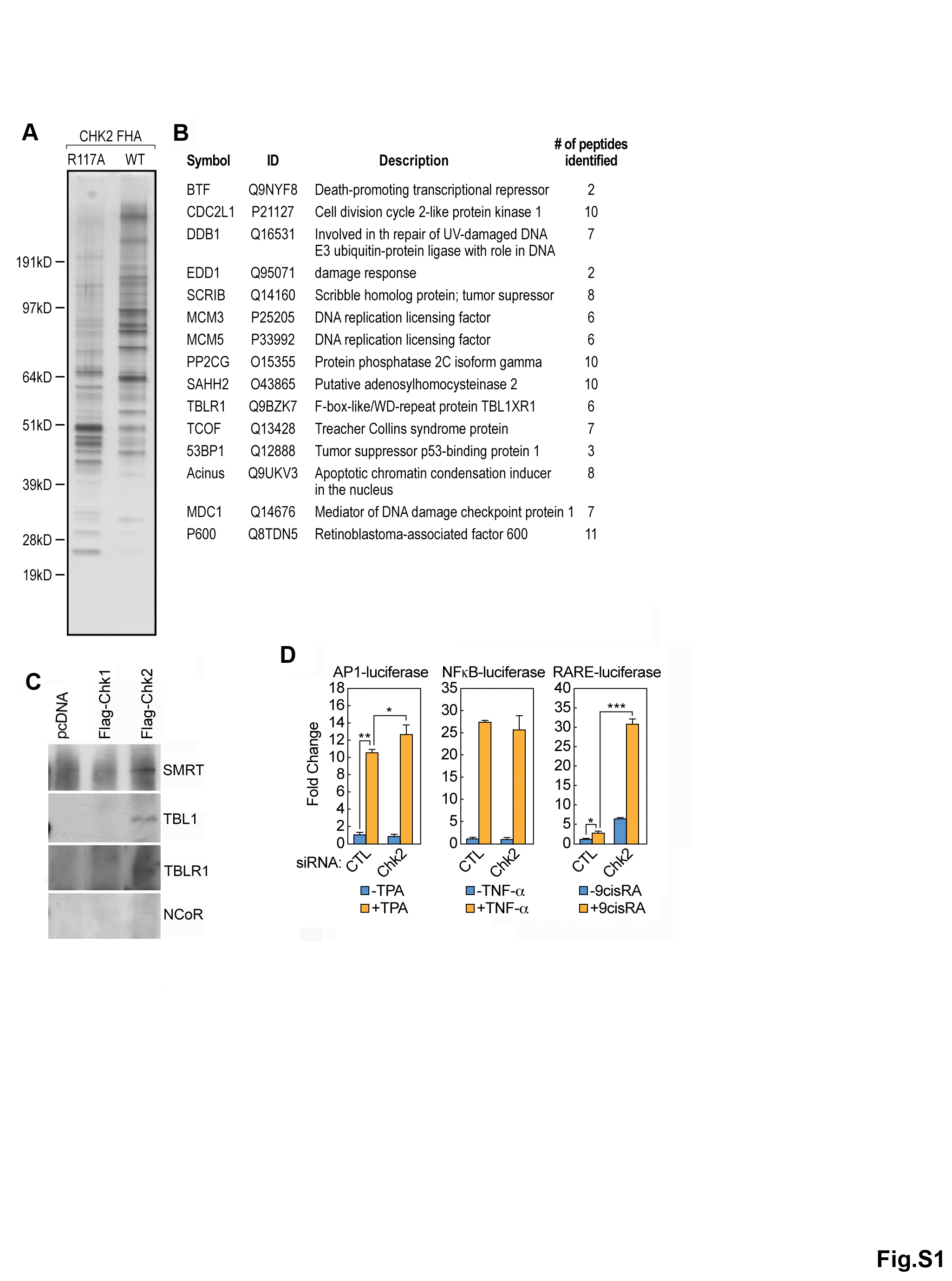

Supplement: Figure S1 — Purification of proteins interacting with the FHA of Chk2. A) A PATH-tagged FHA domain of Chk2 was purified from bacteria and incubated with cellular extracts from HeLa cells. As a control, a mutant lacking the ability to bind target phosphopeptides was used. Purified proteins were run on polyacrylamide gels and visualized with silver stain. B) Proteins identified to specifically interact with the FHA domain of CHK2. C) U2OS cells were transfected with Flag-tagged Chk1 or Chk2 expression vectors, and protein extracts were immunoprecipitated with anti-Flag antibody, followed by SDS-PAGE and Western Blot analysis. D) U2OS cells were transfected with the indicated reporters with or without siRNA against Chk2, incubated for 2 days in serum-free medium and then treated with 10 ng/µl TPA, 20 ng/µl TNF-α or 5×10^−8^ M 9cisRA, as indicated. Statistical significance was calculated on the ratio between luciferase luminescence and Renilla luciferase from three independent experiments, each one including four technical replicates, by Student's T-test. (One star: p-value≤0.05, two stars: p-value≤0.01, three stars: p-value≤0.001). (TIF) [file pone.0059986.s001.tif]

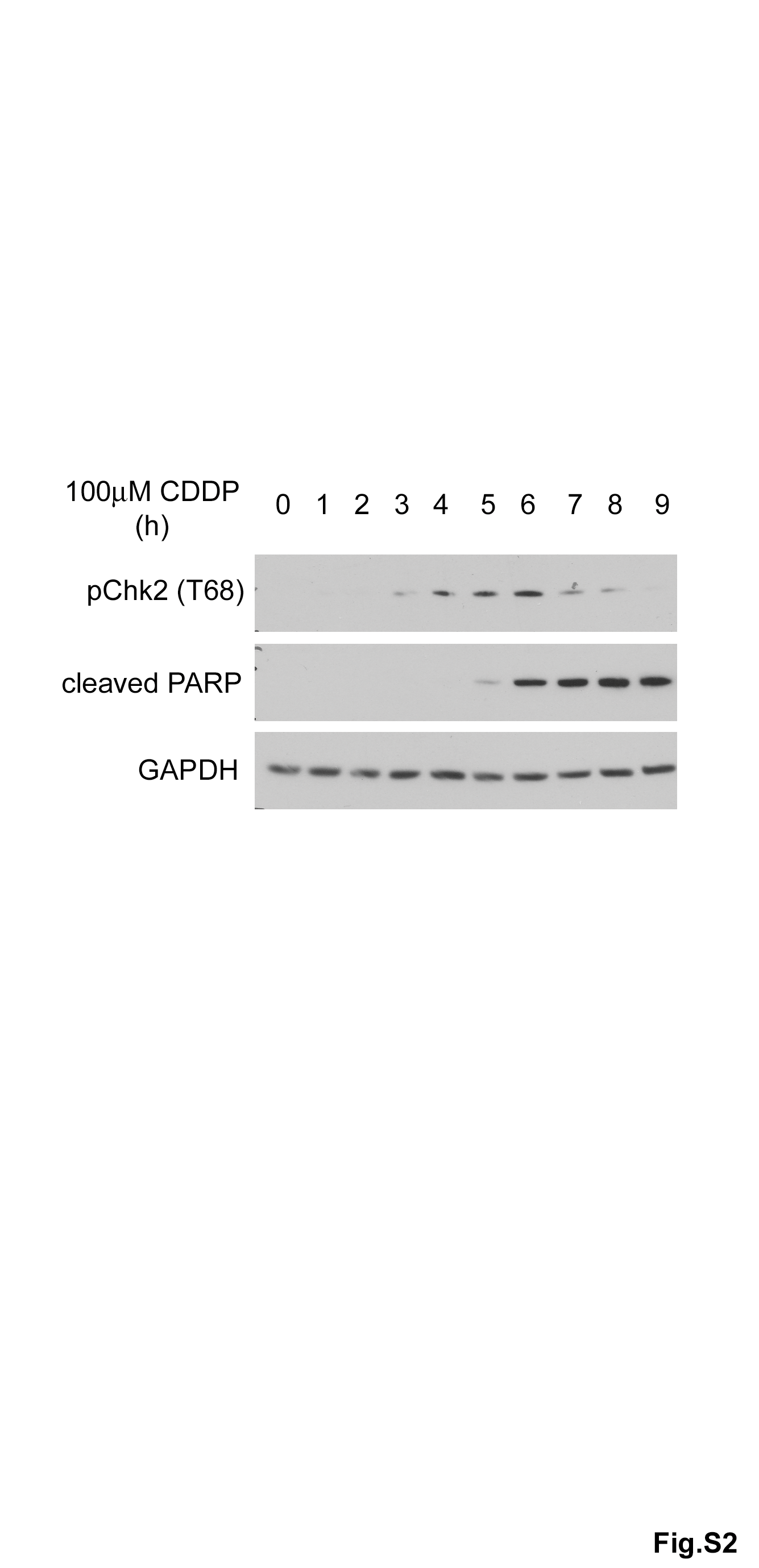

Supplement: Figure S2 — Time-course of cisplatin (CDDP) treatment. U2OS cells were treated with 100 µM CDDP for the indicated time points and the whole cell extracts were used for Western blot with specific antibodies against phospho-Chk2 (T68), cleaved PARP (Asp214), or β-tubulin. (TIF) [file pone.0059986.s002.tif]
