# Supplementary material for: The Co-Repressor SMRT Delays DNA Damage-Induced Caspase Activation by Repressing Pro-Apoptotic Genes and Modulating the Dynamics of Checkpoint Kinase 2 Activation
Source: PLoS One. 2013 May 17;8(5):e59986. doi: 10.1371/journal.pone.0059986 (PMC3656868; doi:10.1371/journal.pone.0059986)
Supplement: Methods S1 — Methods for the data included in Supporting Figures and detailed descriptions of microarray data analysis, plasmids and qPCR primers are included. (DOC) [file pone.0059986.s006.doc]

**SUPPORTING METHODS**

**Plasmids.** DNA fragment encoding human CHK2 FHA (residues 64 to 219) was inserted into pET21-PATH vector (See Complex Purification) using EcoRI and NotI sites. The arginine residue corresponding to CHK2 arginine 117, reported to be crucial for the ability of the FHA domain of CHK2 to bind target phosphopeptides, was mutated to alanine to generate the R117A FHA mutant. Full-length human Chk1 and Chk2 were cloned from U2OS cell-derived cDNA in a pcDNA3 plasmid containing the sequence for Flag-tag at the N-terminus of the insert. The primers used for the PCR reaction are in the Supporting Information. The reporter plasmids TK-AP1-luciferase, TK-NfKB-luciferase, TK-RARE-luciferase, were described earlier [50]. For normalization of luminescence data, we used the Renilla luciferase expression vector pRL-TK.

**Complex purification and MS.** PATH purification was performed as previously described (Smolka et al, J Cell Biol. 2006;175(5):743-53). For cell extract, Hela cells were washed with 10 mM Tris-HCl, pH7.5, 150 mM NaCl, centrifuged and resuspended in TBSN buffer (50 mM Tris-HCl, pH7.5, 10 mM KCl, 10 % glycerol, 2 mM EDTA, 0.2 % NP-40, 1 mM DTT, 1 mM phenylmethylsulfonyl fluroride, 0.2 mM sodium vanadate and 0.5 mM NaF). After incubation on ice for 20 minutes and centrifugation, the supernatant was saved. The pellet was resuspended in TBSN buffer containing 500 mM NaCl, incubated on ice for 20 minutes, and centrifuged. Supernatants were then combined and diluted by equal volume of TBST. Lysate was then split in half for pull down using wild-type FHA domain or the mutant R117A, which has been to shown to not be able to bind target phosphopeptides (Jiejin Li et al, Molecular Cell 2002; 9(5):1045–1054), and bound proteins were eluted as previously described (Smolka et al, 2006).

For quantitative mass spectrometry, proteins eluted from wild-type or R117A pull-down were differentially labeled using light or heavy N-isotag reagent (Smolka et al., Mol Cell Proteomics. 2005; 4(9):1358-69), respectively. Sample were combined and analyzed by uLC-ESI-MS/MS on a quadrupole ion trap mass spectrometer (Finnigan LCQ; Thermo Electron Corporation) as previously described (Smolka et al., 2006). For data analysis, SEQUEST was used for peptide identification, and the XPRESS and INTERACT software were used for quantitation as described previously (Smolka et al., 2005).

**Immunoprecipitation**. U2OS cells were transfected with Flag-tagged Chk1 or Chk2, cell extracts were performed in IPH buffer (50mM Tris-HCl pH 8.0, 150mM NaCl, 5mM EDTA, 0.5% NP-40, 1mM PMSF, 50mM NaF, 2mM Na_2_VO_3_, 1X Complete protease inhibitor mix [Roche] and 1mM glycerol phosphate) and 600μg of protein extracts were incubated with 0.5μg/ml Flag antibody over night, followed by addition of 50 μl protein A sepharose (50% slurry) for each experimental point and incubation for 2 hrs. Immunoprecipitated proteins were washed in wash buffer (same as IPH buffer, but with 0.1% NP-40) 4 times and then resuspended in SDS sample buffer and loaded on acrylamide gel. Western blot was performed with specific antibodies for SMRT and NCoR.

**Luciferase** **assays**. siRNA for Chk2 was transfected in U2OS cells, together with the following reporter plasmids: AP1-, NfKB-, or RARE-TK-luciferase. Cells were starved for 2 days in serum-free medium and then treated for 6hrs with the respective ligands: TPA, TNF-alpha, 9cis-RA. The Renilla luciferase vector pRL-TK was used for normalization. Cellular extracts were performed in 1X Passive lysis buffer (Promega), and luciferase activity was measured by Dual-Luciferase® Reporter Assay System (Promega), following the manifacturer’s instructions. The luminescence in cell extracts was quantified by Veritas microplate luminometer (Promega).

**Microrray data analysis.** Statistical analysis of our microarray experiment proceeded in three steps: 1) low-level analysis of raw data and normalization, 2) sorting of genes according to interest, and 3) statistical analysis of gene ontology terms represented by the sorted list of genes.

**1) Low-level analysis of raw data and normalization.** We calculated the expression levels from scanned images (CEL files) of Illumina BeadChips free academic software *Corgon* (Sasik *et al.*, Bioinformatics. 2002 Dec;18(12):1633-40). This method reduces type-I error rate (false positives), at 1.25-fold level to 4.4%. Genes with *p*-value for presence of 0.1 or less were considered present for discovery purposes. We also imposed an arbitrary but measured condition that only genes whose expression value was greater than 100 at some point in the experiment were considered candidates for further sorting. Expression levels from all experimental conditions were normalized simultaneously using a multi-*loess* technique described in Sasik *et al.* (J Mol Endocrinol. 2004 Aug;33(1):1-9).

**2) Sorting of genes according to interest.** We calculated the absolute and relative changes (with respect to control) of the expression levels for every gene at every time point, and sorted the genes based on their interest statistics. In designing the interest statistic we used the method described in Cole *et al.* (Bioinformatics. 2003 Sep 22;19(14):1808-16), and their software package *Focus.* The interest statistic takes into account not only the fold change in signal levels, but also the initial expression levels of each gene. This choice of weights is designed to sort the genes primarily with respect to their fold changes, but with a secondary consideration for their absolute changes as well.

**3) Statistical analysis of gene ontology terms.** To quantify the statistical significance of a group of genes, we first transformed the integer ranks into real ranks as follows:
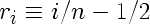
, where *i* is the integer rank of a gene (the first gene on the list has integer rank *n* and a real rank ½). For large *n*, ranks
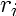
 are quasi-continuous real numbers uniformly distributed between –½ and ½. We next wished to investigate the significance of a group of *m* functionally related genes, whose real ranks were
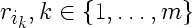
; we defined a rank-sum statistic


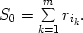


The significance of this group of genes was given by the probability that a score greater or equal to
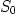
 would be realized for *m* genes that were truly unrelated to the phenotype. To this end we assumed a null model in which the real ranks of the *m* genes were iid (independently and identically distributed) random variables drawn from a uniform distribution on the interval [-½, ½]. For large *m* we used the central limit theorem of statistics in the form.


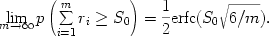


We used the formula on the right-hand side to estimate the *p-*value of a group of genes whenever *m* ≥ 3, and used exact formulas for *m* = 1 and 2. Since a typical pathway involves more than three genes, this asymptotic formula is very accurate.

The group analysis is very powerful, because a group may be statistically significant even when none of its members is particularly highly ranked. This is due to the exponential asymptotic dependence of *p* on *m*:


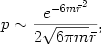


where
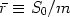
 is the mean rank of the group. It is now easy to see that a large group of genes may be significant even if none of its genes appear among, say, top 100 genes on the sorted list.

**Primers sequences.**

| **Application** | **Forward primer** | **Reverse primer** |
| --- | --- | --- |
| hChk2 cloning | gcactgccgaattctgatgtctcgggagtcg | gcactgccgatatctcacaacacagcagc |
| RAR-β promoter | aatcctgggagttggtgatg | ggcctctgaacagctcactt |
| BCL2L11 RT | tccctacagacagagccaca | tgctcaaggaagagggagag |
| SMAD7 RT | ccgatggattttctcaaacc | ccaggctccagaagaagttg |
| SRPK2 RT | agaggaccctgcggactact | tttcatccaaggctgtctcc |
| FOS RT | gcgtcaacgcgcaggacttct | gctctgtctccgcttggagtgta |
| PPM1D RT | tgatgactccttccccatgt | gggctttagcgcaattttct |
| MAX RT | gccgtaggaaatgagcgata | tgctggtgtgtgtggttttt |
| 18S RT | cgaacgtctgccctatcaact | ctgccttccttggatgtggt |
| FOS promoter | gagcagttcccgtcaatcc | ctgctgacgcagatgtccta |
| IL8 exon 4 | CAGGAATTGAATGGGTTTGC | GGCACAGTGGAACAAGGACT |
